# Retraction statement: Consistency analysis of microRNA‐arm expression reveals microRNA‐369‐5p/3p as tumor suppressors in gastric cancer

**DOI:** 10.1002/1878-0261.13331

**Published:** 2022-12-02

The above article, published online on 04 June 2019 in Wiley Online Library (wileyonlinelibrary.com), has been retracted by agreement between the authors, the journal Editor in Chief, Kevin Ryan, FEBS Press and John Wiley and Sons Ltd. The retraction has been agreed due to the identification of reuse of images from Figs 3 and 4 in at least six other publications, purporting different experiments. Two of these publications came from the same laboratory, and four from other laboratories. Given the extent of the identified issues, the Editors have decided to retract the article.
